# Ultrasound‐Induced Adsorption of Acousto‐Responsive Microgels at Water–Oil Interface

**DOI:** 10.1002/advs.202305395

**Published:** 2023-12-13

**Authors:** Sebastian Stock, Luca Mirau, Matthias Rutsch, Sonja Wismath, Mario Kupnik, Regine von Klitzing, Amin Rahimzadeh

**Affiliations:** ^1^ Soft Matter at Interfaces, Department of Physics Technische Universität Darmstadt Hochschulstraße 8 64289 Darmstadt Germany; ^2^ Measurement and Sensor Technology Technische Universität Darmstadt Merckstraße 25 64283 Darmstadt Germany

**Keywords:** interfacial tension, microgels, ultrasound, water–oil interface

## Abstract

Ultrasonic mixing is a well‐established method to disperse and mix substances. However, the effects of ultrasound on dispersed soft particles as well as on their adsorption kinetics at interfaces remain unexplored. Ultrasound not only accelerates the movement of particles via acoustic streaming, but recent research indicates that it can also manipulate the interaction of soft particles with the surrounding liquid. In this study, it evaluates the adsorption kinetics of microgel at the water‐oil interface under the influence of ultrasound. It quantifies how acoustic streaming accelerates the reduction of interfacial tension. It uses high‐frequency and low‐amplitude ultrasound, which has no destructive effects. Furthermore, it discusses the ultrasound‐induced shrinking and thus interfacial rearrangement of the microgels, which plays a secondary but non‐negligible role on interfacial tension reduction. It shows that the decrease in interfacial tension due to the acoustic streaming is stronger for microgels with higher cross‐linker density. Moreover, it shows that ultrasound can induce a reversible decrease in interfacial tension due to the shrinkage of microgels at the interface. The presented results may lead to a better understanding in any field where ultrasonic waves meet soft particles, e.g., controlled destabilization in foams and emulsions or systems for drug release.

## Graphical abstract

High‐frequency ultrasound accelerates the adsorption kinetics of PNIPAM microgels at water‐oil interfaces leading to a faster decrease in interfacial tension. This acceleration is attributed to the increased transport velocity of microgels facilitated by acoustic streaming. Additionally, PNIPAM microgels are responsive to high‐frequency ultrasound at the interface and thus undergo a volume phase transition, resulting in a reduction in interfacial tension.

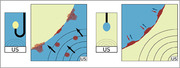

## 1 Introduction

Microgels are soft polymeric cross‐linked particles^[^ ^1^, ^2^ ^]^ which have been investigated for more than three decades due to their broad range of possible applications.^[^ ^3^ ^]^ They are promising drug carriers and release vehicles,^[^ ^4^, ^5^ ^]^ responsive stabilizers of emulsions and foams,^[^ ^6^, ^7^, ^8^, ^9^, ^10^ ^]^ membrane material,^[^ ^11^ ^]^ or coatings.^[^ ^12^, ^13^ ^]^ Especially, the class of stimuli‐responsive microgels draws attention in the soft matter community.^[^ ^14^ ^]^ Poly‐*N*‐isopropylacrylamide (PNIPAM)‐based microgels are the most often studied type of responsive microgels that are able to deform (shrink or swell) in response to outer stimuli. Possible stimuli are for example, changes in temperature, pH value or ionic strength.^[^ ^15^, ^16^, ^17^ ^]^ We recently showed that ultrasound emerges as novel and interesting stimulus for the volume phase transition (VPT) of PNIPAM.^[^ ^18^, ^19^ ^]^ PNIPAM microgels are known for their temperature‐responsive behavior in water, with a volume phase transition temperature (VPTT) of approximately 32 °C,^[^ ^1^ ^]^ the same as lower critical solution temperature (LCST) of linear PNIPAM at low solution concentrations.^[^ ^20^ ^]^ PNIPAM has been studied intensively in bulk and at the interface for more than three decades now.^[^ ^15^ ^]^ In bulk, PNIPAM microgels behave like any other colloidal particles, exhibiting Brownian motion. Their dispersion is stabilized by electrostatic forces, as well as steric interactions, which arise from their charged nature and the dangling chains at their surfaces, respectively.^[^ ^8^ ^]^ At liquid–liquid or liquid–vapor interfaces, microgels adsorb irreversibly and deform strongly due to their softness in a flat shape.^[^ ^21^, ^22^, ^23^, ^24^ ^]^ Most PNIPAM microgels are prepared by precipitation polymerization in a batch approach and posses a manufacturing rooted core‐shell structure. The core is more cross‐linked and therefore stiffer than the less cross‐linked outer shell.^[^ ^25^, ^26^ ^]^ At the interface this structure leads to the well‐known “fried egg‐shape structure” in the adsorbed state.^[^ ^27^ ^]^ It describes the condition in which the soft shell of microgels are more stretched than the rigid core. Overall, the larger proportion of PNIPAM microgels protrudes into the water phase. The characterization of the VPT process at interfaces/surfaces is still under investigation. Recently Vialetto et al.^[^ ^27^ ^]^ made a huge progress in the in situ characterization of the microgel structure at the water–oil interface by Atomic Force Microscopy (AFM). These results confirm earlier studies claiming that most of the VPT‐induced shrinking takes place in the part of the microgel that is protruding into the water phase. Lateral shrinking of the microgels at the interface seems to play a minor role. Pendant drop tensiometry is one method used to characterize the reduction of interfacial tension resulting from the adsorption of microgels at the interface. With this technique, the interfacial tension of a pendant or rising drop is measured by analyzing its shape. This method allows for the time‐resolved measurement of surface/interfacial tension and the monitoring of the adsorption process of the collective microgels. Li et al.^[^ ^28^, ^29^ ^]^ and Tatry et al.^[^ ^30^, ^31^ ^]^ studied the influence of various parameters, e. g., temperature, cross‐linker density and concentration of microgel dispersion, on the adsorption kinetics of PNIPAM microgels at the interface of a pendant drop. They showed that the decrease in interfacial tension is initially diffusion‐driven but becomes adsorption limited for the later saturation behavior. Thus, the reduction in interfacial tension accelerates with increasing concentration and with decreasing cross‐linker density, both affecting the coverage area of the microgels at the interface. The temperature influences the properties of the liquid, as well as induces a VPT in the microgels, affecting the size and charge of the microgels. This leads to a nonlinear behavior of the adsorption time. Below the VPTT, an increase in temperature increases the diffusivity of the microgels toward the interface. Additionally, it facilitates the adsorption process of microgels in the adsorption limited regime. This is expressed by the reduced final reachable value (steady‐state) for the interfacial tension. Above the VPTT, the increase in charge density of the microgels hinders the adsorption of more microgels. This reduces the adsorption speed and increases the steady‐state interfacial tension.

However, temperature is not an effective stimulus to control the adsorption of microgels at interfaces due to its limited versatility. The response time of the whole system including sub‐phase and cell is slow. Furthermore, the required amount of energy input for steady‐state condition is high. Making use of ultrasound as a control parameter offers several advantages such as short response time, fast switch‐ability and a comparably low energy input. In particular, being able to induce the VPT of microgles by ultrasound at the interface may lead to novel applications in various fields, e.g., pharmacology, food industry or interfacial catalysis.^[^ ^19^, ^32^ ^]^ By shrinking microgels after adsorption, embedded payload material can be released at the interface, allowing for controlled and targeted delivery or catalysts. It has to be emphasized that here ultrasound is not destructive since it has much higher frequencies (MHz range) and lower power amplitudes (W range) compared to typical ultrasound baths that operate in kHz range with power amplitude in the range of hundreds of Watts.

When imposing ultrasound on a liquid the waves propagate through the medium and the acoustic energy attenuates due to the liquid viscosity. Due to nonlinearities a flow is generated that is called acoustic streaming.^[^ ^33^, ^34^ ^]^ In particle dispersions, the acoustic radiation force can lead to different acoustophoretic velocities depending on the particle size and position^[^ ^35^ ^]^; i. e.,

(1) $$v_{s}=\frac{2\Phi ka^{2}I}{3\eta }\sin\left(2kz\right)$$

where Φ is a contrast factor related to the compressibility of the particles with respect to the compressibility of the solvent, *k* is the wave number, *a* is the particle diameter, η is the viscosity of the dispersion and *z* is the particle position. Having the acoustic energy density, *I* = *P* ^2^/2ρ*c* ^2^, related to acoustic pressure *P*, liquid density ρ, and speed of sound *c* in the liquid, Equation (1) becomes

(2) $$v_{s}=\frac{\Phi ka^{2}P^{2}}{3\eta \rho c^{2}}\sin\left(2kz\right)$$

From Equation (2) one can infer that the ultrasound induces a flow having a maximum velocity of $\frac{\Phi ka^{2}P^{2}}{3\eta \rho c^{2}}$. This flow may affect the adsorption kinetics of particles at the interface.

In the present study, we investigate the influence of ultrasound on the adsorption kinetics of microgels at the water‐oil interface. The objective of this study is to identify the factors that influence the acceleration of adsorption and to investigate the potential VPT of the microgels induced by ultrasound at the interface. For this purpose, we synthesized microgels with different cross‐linker densities, i.e., stiffnesses. Furthermore, we modified the pendant drop technique to enable the measurement of the interfacial tension in the presence of ultrasound.

## 2 Results

### 2.1 Features of the Modified Pendant Drop Device

**Figure 1:**
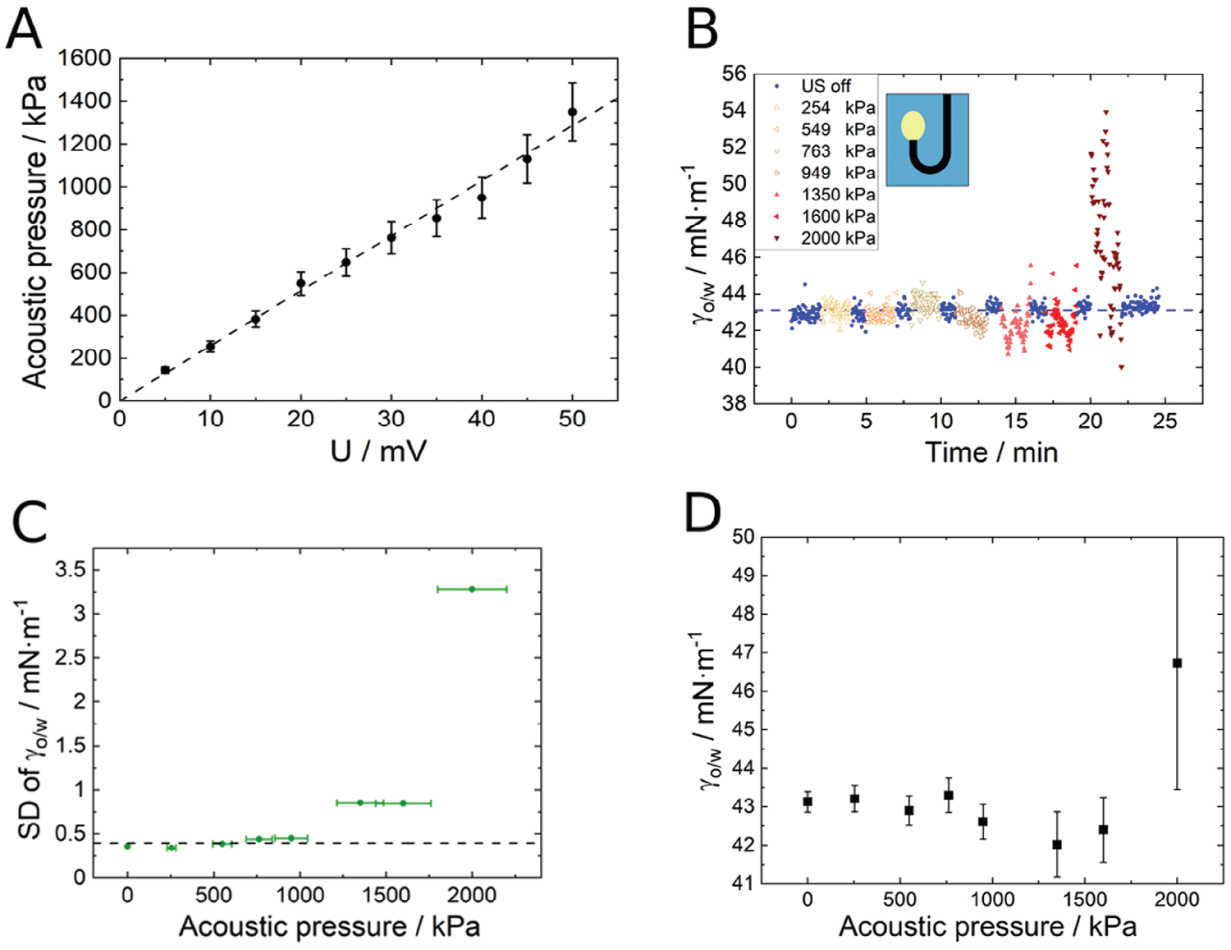
A) The acoustic pressure over the applied voltage at the signal generator. B) The influence of ultrasound on the measured interfacial tension values (γ_o/w_). A rising dodecene drop in water was utilized for this test. C) The standard deviation (SD) of the interfacial tension against the acoustic pressure calculated for the measurements shown in (B). D) Mean values of interfacial tension at different acoustic pressures. The error bars were calculated from the standard deviation of multiple measurements.

The results of the technical test of the setup allowing to measure the interfacial tension under ultrasound are shown in **Figure** **1**. There is a linear dependency of the measured acoustic pressure over the set voltage amplitude at the signal generator (Figure 1A). This plot shows that the acoustic pressure can be adjusted by controlling the input voltage at the signal generator. In addition, the distortion of the measured interfacial tension γ_o/w_ due to the influence of ultrasound was quantified (Figure 1B,C). The ultrasound is turned off and on again in order to determine the effect on the measured value for γ_o/w_. At each step, the acoustic pressure is increased. The higher the acoustic pressure amplitude, the more it impairs the quality of the measurement data. The scattering of the data points characterized by their standard deviation (Figure 1C) increases significantly above around 1000 kPa. Below this value, a reasonable measurement is possible. The systematic shift in apparent average interfacial tension is negligible for lower acoustic pressures, but becomes pronounced at higher values, interfering significantly with the measurement accuracy. At acoustic pressures below 1000 kPa, the standard deviation of the measured values is in the range of usual measurements (≈ 0.3 $\frac{\text{mN}}{m}$). Therefore, we use acoustic pressure below 1000 kPa in this study. Note that for the generation of the highest pressures 1600 and 2000 kPa Figure 1B, the data in Figure 1A were linearly extrapolated and the corresponding voltage was applied. At high pressures the disturbance of the liquid interface was too strong to get reliable values for the interfacial tension (Figure 1B,C). Destructive effects usually occur at higher ultrasonic intensities, well into the megapascal (MPa) range and beyond, leading to cavitation or droplet breakup. Our results suggest that ultrasound is not destructive below 800 kPa as indicated by consistent interfacial tension values, and we've measured a minimal temperature increase (2°C) at 763 kPa, where cavitation is unlikely at MHz‐range frequencies.

### 2.2 Adsorption Kinetics of the Microgels

Detailed temperature‐dependent swelling and shrinking curves of microgels in water were measured by dynamic light scattering (DLS) and are presented in the Figure S3 (Supporting Information). For further information about the microgels please check our previous publications.^[^ ^7^, ^9^ ^]^ The microgels have a diameter of around 600–750 nm and around 250–300 nm below and above their VPTT, respectively. The microgels are positively charged due to the positively charged starter AAPH. Their charge density increases at elevated temperatures above the VPTT. The swelling ratio ($R_{\text{H, swollen}}^{3}/R_{\text{H, shrunken}}^{3}$) decreases with increasing cross‐linker density from around 20 to around 5. As known from literature, the higher cross‐linked microgels are stiffer than the lower cross‐linked ones.^[^ ^10^ ^]^ Softer microgels exhibit greater interfacial extension, allowing them to cover larger areas at the interface compared to their stiffer counterparts. Consequently, achieving a reduction in interfacial tension requires a lower quantity of microgels, resulting in a faster reduction of interfacial tension.^[^ ^28^, ^29^ ^]^

**Figure 2:**
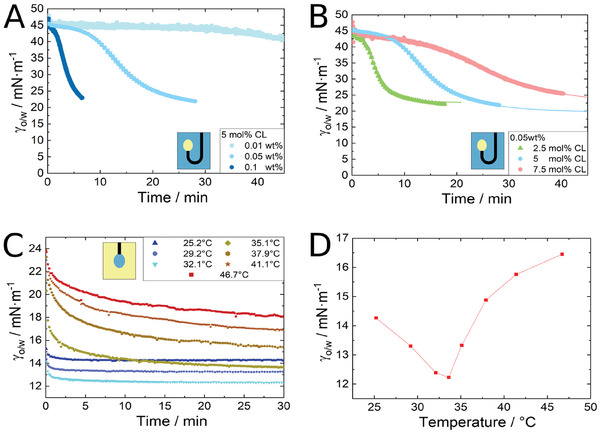
Influence factors on the adsorption kinetics of microgels: Decrease in interfacial tension of a rising dodecene drop in an aqueous microgel dispersion A) due to the adsorption of the medium cross‐linked microgels (5 mol.% cross‐linker) at different concentrations, B) with increasing cross‐linker density. C) The time‐resolved interfacial tension of a pendent aqueous drop of the lower cross‐linked microgels (2.5 mol.% cross‐linker) in oil at different temperatures. D) Final values of the interfacial tension for the medium cross‐linked microgels (5 mol.% cross‐linker) with increasing temperature.

**Figure 3:**
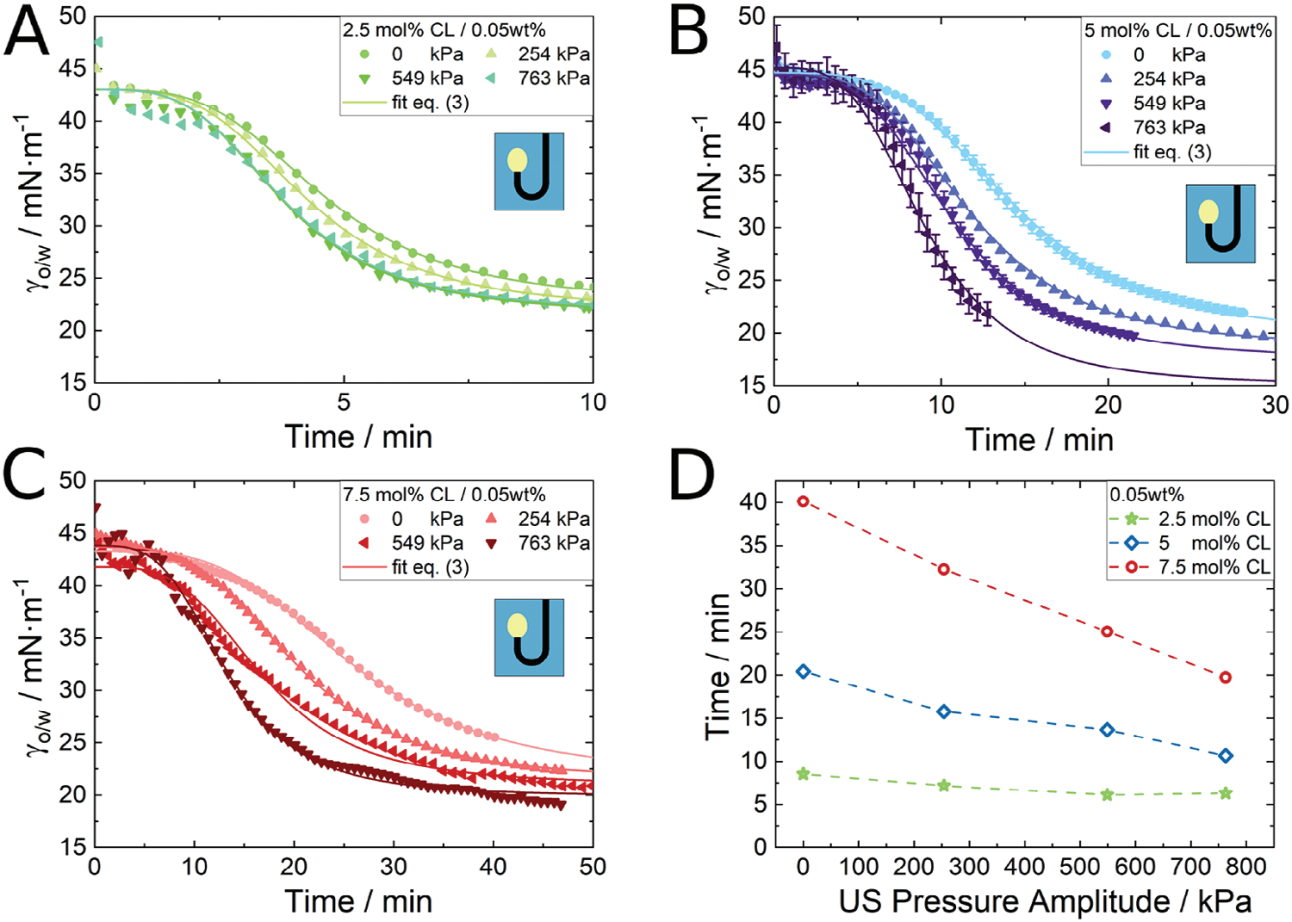
Decrease in interfacial tension of a rising dodecene drop in an aqueous microgel dispersion due to adsorption of A) 2.5 mol.% cross‐linker, B) 5 mol.% cross‐linker, and C) 7.5 mol.% cross‐linker microgels at the interface subjected to ultrasound. D) Time to reach γ_o/w_=25 mN·m^−1^ for the different microgel systems over the applied pressure amplitude.

**Figure** **2** shows the adsorption kinetics of microgels at interface without ultrasound. The usage of different configurations (rising or pendant drop) is justified in the experimental section (Section Tensiometry Device Subjected to Ultrasound (Pendant/Rising Drop)). Figure 2A,B show the interfacial tension reduction due to the adsorption of microgels at the interface of a rising oil drop at different microgel concentrations and cross‐linker densities of the microgels, respectively. A higher concentration as well as a lower cross‐linker density accelerates the decrease of γ_o/w_. Characteristic kinetics of interfacial tension reduction includes three consecutive regimes: 1‐ no/minor change in γ, 2‐ strong change in γ and 3‐ no/minor change in γ. The transient interfacial tension can be described empirically by^[^ ^36^ ^]^

(3) $$\gamma \left(t\right)=\gamma_{\infty }+\frac{\gamma_{0}-\gamma_{\infty }}{1+{\left(t/\tau_{d}\right)}^{n}}$$

where γ_0_ and γ_∞_ are initial and equilibrium interfacial tensions, respectively and τ_*d*_ is the characteristic time for the interfacial tension reduction and *n* is the fit parameter (which was best fitted to 3.5 here). Using Equation (3), we fitted the results from **Figure** **3**B for three different cross‐linker contents. The diffusion and adsorption characteristic time for 2.5, 5, and 7.5 mol.%, respectively are 285.5, 835.6, and 1604 s.

Figure 2C, D show the effect of temperature on the interfacial tension reduction of a pendant microgel dispersion drop. Changing the temperature leads to a non‐monotonous change in the decrease of γ_o/w_: According to the Figure 2C, increase from 25 to 32 °C the decrease in γ_o/w_ is faster than increase from 32 to 47 °C. Around the VPTT the kinetic is the fastest (Figure 2C) and the steady‐state value of interfacial tension reaches a minimum in the final value (Figure 2D).

Ultrasound has a strong effect on the time‐resolved interfacial tension reduction of the water‐oil interface due to the adsorption of microgels for all three different cross‐linker densities (Figure 3A–C). The microgel concentration was fixed at 0.05 wt%, and ultrasound accelerates the decrease in interfacial tension. The data indicate that the final value with ultrasound is lower than the one without ultrasound. The use of ultrasound during the interfacial tension measurement poses a challenge as it increases the risk of drop rip‐off. This was especially the case at low interfacial tensions in the later stages of the measurement. Figure 3D shows the time needed for the interfacial tension to decrease to 25 mN·m^−1^. This value was chosen, because it is the lowest interfacial tension, that was achievable in all measurements and attributed to the first two regimes mentioned in Section 2.2. This evaluation should give a rough estimation of the quantitative adsorption acceleration induced by ultrasound. According to Figure 3D, the needed time for decreasing the surface tension to 25 mN·m^−1^ decreases linearly with increasing the applied ultrasound pressure.

### 2.3 Effect of Ultrasound on Adsorbed Microgels at the Interface

**Figure 4:**
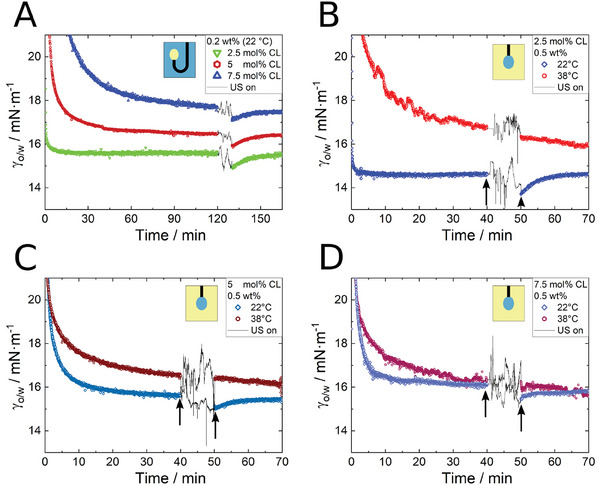
Interfacial tension γ_o/w_ A) for a dodecene drop in an aqueous microgel (in different cross‐linker densities and a fixed concentration of 0.2 wt%) dispersion below VPTT (22 °C), and B–D) for a drop of aqueous microgel (in different cross‐linker densities and a fixed concentration of 0.5 wt%) dispersion in dodecene, below and above the VPTT. After the equilibration time in all cases, the drop was exposed to ultrasound for 10 min. (Applied ultrasound sound pressure amplitude: 763 kPa)

To investigate the influence of ultrasound on the microgels that were already adsorbed at the interface, we initially waited for 120 min in case of the oil drop in water and 40 min in case of a water drop in oil to allow the microgels to adsorb at the interface before imposing ultrasound. The concentration of microgels in the aqueous phase for the rising drop experiment was fixed at 0.2 wt% which is lower than the concentration for the pendant drop experiment (0.5 wt%). However, at 0.5 wt% and above, the increasing turbidity of the outer phase prevented accurate measurements. After the respective equilibration time, the microgel‐laden drops were subjected to ultrasound for 10 min in order to observe the effects. **Figure** **4** shows the resulting interfacial tension, γ_o/w_, on the respective drops over time. In both cases, rising drop or pendant drop, below VPTT (22 °C) the respective equilibration time was enough to saturate the interface with microgels. Below VPTT, ultrasound causes a decrease in interfacial tension γ_o/w_, lower than its former value, and even lower than any steady state values measured without ultrasound. Upon turning off the ultrasound, the interfacial tension returns to the former steady‐state value in a relaxation‐like manner. This phenomenon becomes less pronounced as the cross‐linker density increases. Figure 4B–D show the results for a water drop in oil at different microgel cross‐linker densities, both above and below VPTT. The microgels concentration was fixed now at 0.5 wt% in order to prevent depletion effects of microgels in the droplet. Below the VPTT, the same phenomenon occurs as observed in the case of rising oil drop in water shown in Figure 4A. Above the VPTT, the interfacial tension γ_o/w_ appears to be almost unaffected by the ultrasound. After turning off the ultrasound, γ_o/w_ continues along the expected data curve. No ultrasound‐induced decrease can be observed apart from the expected adsorption process.

**Figure 5:**
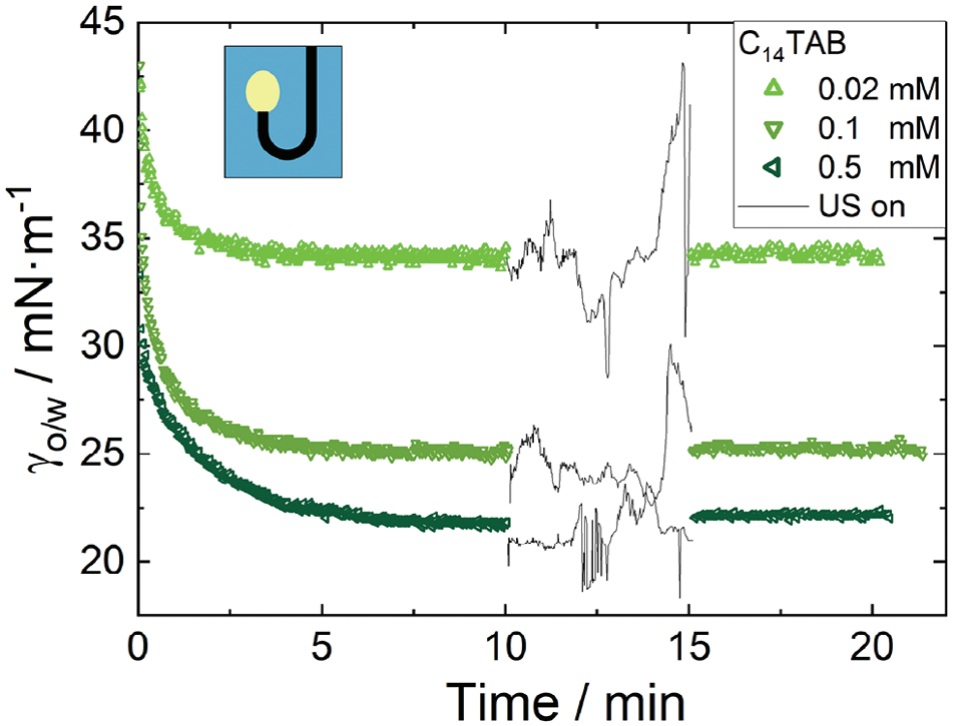
Influence of ultrasound on the interfacial tension of a rising drop of dodecene in an aqueous solution of a classical surfactant (C_14_TAB) at three different concentrations below the critical micellar concentration.

A possible explanation for the changes in interfacial tension upon imposing ultrasound is the capability of the ultrasound to desorb or increase the adsorbed amount of microgels at the interface. In order to distinguish between the adsorption and other microgel specific processes that affects the interfacial tension a drop covered with a classical surfactant was studied. The adsorption/desorption energy of low molecular weight surfactants is significantly lower (several *k*_B_ *T*) than that of microgels (up to several thousand *k*_B_ *T*).^[^ ^37^ ^]^ If the ultrasound were able to influence the balance of adsorption or desorption of the surfactant, it would result in a change in the interfacial tension after exposure to ultrasound. However, as **Figure** **5** shows, this is not the case. After turning off the ultrasound, the interfacial tension of the drop returns immediately to the same value as γ_o/w_ before the ultrasound treatment. This means that the ultrasound does not affect the adsorption/desorption of surfactant at the interface. Therefore, the ultrasound‐induced interfacial tension reduction in case of microgels has another explanation which is discussed below.

## 3 Discussion

We used pendant/rising drop tensiometry to characterize the influence of ultrasound on the adsorption kinetics (or generally, interfacial tension reduction) of microgels at the water‐oil (1‐dodecene) interface. As a first step, it is important to test the setup and quantify the influence of ultrasound on the quality of the measured values of interfacial tension. Figure 1 demonstrates that ultrasound has a negligible influence on the quality of the measured data as long as the sound pressure is below 1000 kPa. This qualifies the setup to investigate the adsorption behavior of microgels under the influence of ultrasound. The microgels were produced with various cross‐linker densities, which correspond to different levels of stiffness. Consistent with well‐established studies by Tatry et al.^[^ ^31^ ^]^ and Li et al.^[^ ^28^ ^]^ we identify dispersion concentration, cross‐linker density and temperature as main factors that influence the reduction of interfacial tension by microgels (Figure 2). At higher concentrations more particles reach the drop interface within the same time interval. This leads to a faster decrease in interfacial tension. The cross‐linker density influences the adsorption kinetic based on their microstructure and mechanical properties. Lower cross‐linker density enhances the deformability of microgels, enabling them to cover a large interfacial area with the same particle quantity. The higher deformability and the existence of more dangling chains facilitate their adsorption at the interface leading to a faster interfacial tension reduction. Temperature has two counteracting effects on interfacial tension of a microgel dispersion. On one hand, elevated temperatures result in an augmented diffusion coefficient for microgels, attributed to a reduced viscosity of the surrounding liquid and enhanced particle mobility. This again leads to a decrease in adsorption time. On the other hand, the temperature‐induced shrinking of microgels around the VPTT decreases the covered area per microgel and increases the charge density of the microgels. Therefore, the adsorption of additional microgels at the interface becomes less likely leading to slower interfacial tension reduction. The steady‐state value of the interfacial tension goes through a minimum around the VPTT of the microgels (Figure 2D). These findings are in good agreement with previous studies by Li et al.^[^ ^28^, ^29^ ^]^ and are explained by an increased deformability of the microgels around the VPTT.

**Figure 6:**
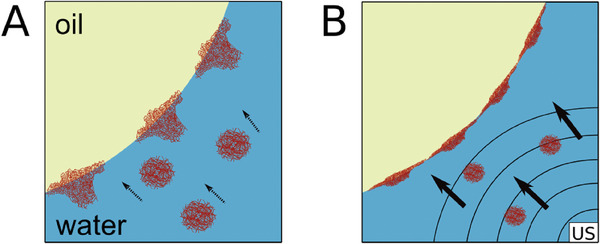
Depiction of the adsorption process of microgels at the water‐oil interface with and without US. A) Without US microgels are able to spontaneously adsorb at the water‐oil interface in a deformed state. B) US induces acoustic streaming in the surrounding water of the droplet and pushes the US‐induced collapsed microgels toward the interface. This reduces the time to reduce the interfacial tension.

Our results identify ultrasound as an additional factor affecting the adsorption kinetics of microgels (Figure 3). The reduction in interfacial tension speeds up with increasing the acoustic pressure. The ultrasound‐induced reduction in interfacial tension can be attributed to two distinct and independent effects, as it is schematically described in **Figure** **6**. The first effect arises from the influence of ultrasound on the transport velocity of microgels toward the interface (the bold arrows in Figure 6B). The faster transport toward the interface is largely explained by acoustic streaming. According to Equation (2), in our experiments the order of magnitude of the streaming velocity can be estimated^[^ ^18^ ^]^ to 0.1 $\frac{\text{mm}}{s}$ since $k\approx O(10^{4}\;m^{-1})$, $a^{2}\approx O\left(10^{-13}\;m^{2}\right)$, $P^{2}\approx O\left(10^{12}\;N^{2}m^{-4}\right)$, $\eta \approx O(10^{-3}\;Nm^{-2}s)$, $\rho \approx O(10^{3}\;kgm^{-3})$, and $c^{2}\approx O\left(10^{6}\;m^{2}s^{-2}\right)$. The contrast factor Φ for PNIPAM microgels can be estimated in the order of 0.1 according to some measurements for other polymers and soft particles.^[^ ^38^, ^39^ ^]^ By defining a characteristic length *l* ≈ 30 mm (the liquid height in the cuvette), the streaming characteristic time τ_*s*_ = *l*/*v*_*s*_ can be estimated to about 5 min. The choice of this specific length is driven by the collective behavior of particles as they navigate through the geometry to reach the oil‐water interface. We are particularly interested in understanding how quickly acoustic streaming influences the transport of microgels to the interface within this defined distance from the bottom to the top. Altering the cuvette size would substantially impact this time frame. These estimations provide a better understanding of the influence of the acoustic streaming on microgel dispersions at different cross‐linker densities. For example, for the cross‐linker density of 2.5 mol.%, τ_*s*_ is close to the characteristic time of interfacial tension reduction, τ_*d*_. The interfacial tension reduction itself is fast enough that the acoustic streaming does not change it substantially. This is because the microgel deformability is high enough that the interfacial tension reduction is mainly controlled by deformation and rearrangement of microgels at the interface rather than being transport‐controlled. Therefore, the acoustic streaming which mainly affects the transport velocity of microgels does not influence the interfacial tension reduction of soft microgels significantly (at high concentrations). The streaming effect becomes more pronounced with increasing cross‐linker density, as the τ_*d*_ of microgels increases, leading to the domination of the streaming as the main time‐limiting factor of interfacial tension reduction. The second effect arises from the volume phase transition of microgels due to the ultrasound (the collapsed microgels in bulk and at the interface). In previous studies,^[^ ^18^, ^19^ ^]^ we demonstrated that the absorbed energy from ultrasound by the liquid is able to break hydrogen bonds between linear PNIPAM and surrounding water molecules. In the current study, it appears that the PNIPAM microgels are responsive to ultrasound as well. We showed this by illustrating the surface tension reduction lower than the steady‐state value upon imposing ultrasound to a drop fully covered by swollen microgels (Figure 4). A comparison with a drop covered with surfactants (Figure 5) reveals that the further interfacial tension reduction (observed in the case of microgels) below the steady‐state value is only attributed to the VPT of microgels. The energy provided by the ultrasound is not able to detach the microgels from the interface. The reason behind this claim is in twofold: First, desorption of microgels from the interface would result in an increase in interfacial tension rather than a decrease. Second, the absence of any effect on the measured interfacial tension of the drop covered with the surfactant upon imposing ultrasound shows that there is no desorption/adsorption of the surfactants. Since the system with the classical surfactant has a significantly lower interfacial adsorption energy compared to the microgels, we can rule out the desorption of microgels due to ultrasound, as microgels have a much higher adsorption energy.

**Figure 7:**
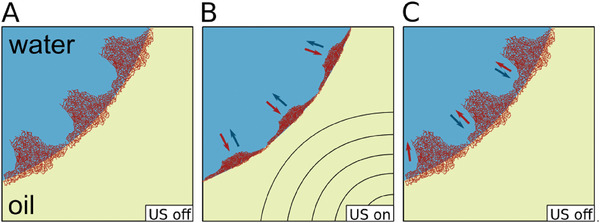
Proposed model to describe the behavior of microgels at the oil water interface under the influence of ultrasound. A) Ultrasound off: microgels adsorb spontaneously at the interface of the drop and reduce the interfacial tension until the drop is fully covered. B) Ultrasound on: Under the influence of ultrasound the microgels are shrinking mostly horizontally to the interface and the water is pushed away from the interface. The interfacial tension decreases. C) After ultrasound is turned off the microgels relax in their initial state similar to (A). The interfacial tension also relaxes to the initial value. The blue arrows indicate the direction of the water and red arrows show the direction of shrinking/swelling of microgels.

The following model explains the unexpected decrease in interfacial tension caused by ultrasound: By applying ultrasound to a fully microgel‐covered water drop in oil (**Figure** **7**A), the hydrogen bonds in the part of the microgels that is protruding in the water phase break and the microgels shrink (Figure 7B). This leads to the compression of the microgels against the interface and discharging water from the interface leading to the reduction of interfacial tension. Depending on the elasticity of the microgels, the shrinking capability, and, thus, the subsequent changes in interfacial tension vary. For instance, softer microgels undergo more significant shrinkage leading to a greater interfacial tension reduction (Figure 4). By turning off the ultrasound, the hydrogen bonds reform, the microgels relax, start reswelling and uptaking water again (Figure 7C). The interfacial tension returns to its steady‐state value. This effect occurs at interfaces of both oil droplet in water and water droplet in oil.

The effects of ultrasound and temperature on the interfacial tension reduction of the water–oil interface due to microgel adsorption share similarities and differences. Both parameters increase the transport velocity of microgels toward the interface leading to an accelerated reduction in interfacial tension. Temperature performs this by enhancing the diffusion, and ultrasound achieves it by introducing acoustic streaming. In addition, both parameters induce a VPT of the microgels at the interface leading to changes in final interfacial tension value. However, in case of temperature, the interface experiences a minimum interfacial tension around the VPTT while in case of ultrasound the final value of interfacial tension always reduces by increasing the acoustic pressure. This leaves rooms for further investigations in future. Furthermore, while temperature needs a long time to be steady and homogeneous through the liquid, ultrasound is a fast stimulus that can be quickly switched on. This characteristic of ultrasound could be an important asset for various applications.

## 4 Conclusion

In this study, for the first time we have introduced the ultrasound‐induced adsorption of microgels at the water‐oil interface. High‐frequency and low‐amplitude ultrasound in contrast to regular sonication systems is not destructive. We conducted a systematic investigation by studying the reduction in interfacial tension of a drop due to the adsorption of microgels. We show that for the applied ultrasound frequency and the investigated ultrasound amplitudes the technique of pendant/rising drop tensiometry is still applicable and gives new insights on microgels adsorption. Imposing ultrasound, generally enhances the transport velocity of the microgels toward the interface through the acoustic streaming. As a result, ultrasound reduces the required time for the interfacial tension to reach its steady‐state value. This effect is more pronounced in cases where their interfacial tension reduction is mainly diffusion‐controlled, e.g., at higher cross‐linker densities (5 and 7.5 mol.%) and fixed dispersion concentration (0.05 wt%). Softer microgels (at this concentration) exhibit rapid tension reduction even without ultrasound, while stiffer microgels benefit more from the acceleration provided by acoustic streaming due to ultrasound.

Furthermore, we found that ultrasound induces a VPT of the microgels at the interface that results in a further decrease in the final interfacial tension value. This effect also depends on the mechanical properties of the microgels. Softer microgels tend to have a higher swelling ratio, meaning they undergo more significant size reduction upon ultrasound stimulation. This leads to a stronger reduction in interfacial tension.

While this ultrasound‐induced VPT may have a relatively minor impact on the observed interfacial tension, it presents significant potential for various applications such as cargo transport toward or drug release at interfaces. In future studies, exploring the possibility of delivering drug‐loaded microgels at interfaces and triggering drug release through ultrasound stimulation can be a promising avenue to pursue. This approach harnesses the unique capabilities of microgels and ultrasound to enable targeted and controlled drug delivery at interfaces, opening up new opportunities in the field of therapeutic interventions.

## 5 Experimental Section

### Material

Purified water with a specific resistance of 18.2MΩ ·cm at 25 °C was used from a Milli‐Q purification system (Merck KGaA, Germany). The oil 1‐dodecene (>96%), *N*‐isopropylacrylamide (NIPAM) and *N*,*N*'‐methylenbisacrylamid (BIS) were purchased from Sigma–Aldrich (Merck KGaA, Darmstadt, Germany). 2,2'‐azobis‐2‐methyl‐propanimidamide dihydrochloride (AAPH) was purchased from Cayman Chemical Company (Cayman Chemical, USA).

### Microgel Synthesis and Cleaning

Microgel particles (microgels) were synthesized using a common and broadly used recipe via precipitation polymerization reaction.^[^ ^1^, ^16^, ^40^, ^41^, ^42^ ^]^ NIPAM (monomer) and BIS (cross‐linker) were dissolved in 120 ml water. The solution was degassed under constant stirring (1000 RPM) and a constant nitrogen flow through the solution for at least 1 h at 80 °C. To achieve a variation in cross‐linker density, the amount of cross‐linker was varied while the sum of NIPAM and BIS molecules was kept constant at 0.02 mol. The given cross‐linker content is the molecular fraction of BIS to the total amount of NIPAM and BIS molecules. The microgels were synthesized with three different cross‐linker densities: lower cross‐linked microgel (2.5 mol.% BIS), medium cross‐linked microgel (5 mol.% BIS) and higher cross‐linked microgel (7.5 mol.% BIS). 33.5 mg AAPH was used as an initiator. AAPH was dissolved prior to injection in 1 ml water and injected via a syringe. The reaction was then carried out for 90 min at 80 °C and 1000 RPM. The obtained microgels were cleaned primarily by dialysis for at least ten days (ten cycles, 120 ml dispersion against 50 l water in total). In addition, the microgels were cleaned by sedimentation during centrifugation (10.000·g, 30 min) and subsequent redispersion. This cleaning procedure was repeated at least four times. The concentration after redispersion was checked by drying three samples of 1 g of the dispersion in an oven (60 °C, 1 hour) and weighing the residual microgel. The value was obtained by averaging over these three samples. After each step the concentration was adjusted to 0.05 wt.% and the purity of the microgel dispersion was checked via pendant drop tensiometry against air. The dispersion was considered as clean when the subsequent cleaning step did not change the surface tension decrease, i.e., it did not result in any changes in the surface tension over time curves in comparison to the previous step (Figure S1, Supporting Information). After cleaning, the microgels were dried by freeze‐drying and stored at ‐20 °C until usage.

### Microgel Characterization

The microgel (0.006 wt%) sizes were measured with DLS to obtain the hydrodynamic diameter with a DLS setup from LS instruments (Switzerland). The microgels ζ‐potential was measured with a Zetasizer nano from Malvern Panalytical (United Kingdom). For a detailed description of the characterization see our previous publications.^[^ ^7^, ^9^ ^]^

### Tensiometry Device Subjected to Ultrasound (Pendant/Rising Drop)

**Figure 8:**
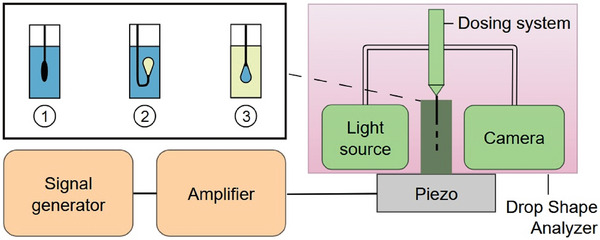
Depiction of the used setup for drop shape analysis under the influence of ultrasound. The piezo crystal was glued to a cuvette and was operated via a signal generator and an amplifier. 1) For the acoustic pressure measurements the dispensing tip was replaced with a hydrophone. For the interfacial tension measurements either 2) rising drop (of 1‐dodecene) or 3) pendant drop technique was used.

Interfacial tension measurements were carried out with a drop shape analyzer OCA 20 (DataPhysics instruments, Filderstadt, Germany). The device was modified, by adding an ultrasound transducer under the cuvette, for measurements of the interfacial tension under the influence of ultrasound. The setup is depicted in **Figure** **8**. More information and photos are given in the Figure S2 (Supporting Information). A quartz glass cuvette (inside dimensions 10mm x 10mm x 40mm, Hellma Analytics, Müllheim, Germany) was attached to a piezo‐ceramic ultrasonic transducer (resonance frequency 2.34 MHz, SOAR Tech., Shenzhen, China) using a two component latex glue (UHU Endfest Plus300, Bühl, Germany). In order to hold the transducer with the attached cuvette a holder was designed and 3D printed. A function generator (SDG1062X, SIGLENT, Shenzhen, China) was used to generate a sinusoidal signal with a frequency of 2.34 MHz, which was amplified by an RF amplifier (VBA100‐30, Vectawave, Newport, UK). The resulting acoustic pressure in the cuvette was measured in a separate measurement by replacing the dispensing tip with a needle‐shaped hydrophone (HNR‐1000, Onda, Sunnyvale, USA). The ultrasonic pressure was measured in a way that the sensitive plane of the hydrophone needle and the piezo‐surface were parallel to each other. The tip of the needle was placed at the position in the cuvette where the drop was generated later. microgel adsorption kinetics with and without ultrasound were measured by filling 3.25 ml microgel dispersion of 0.05 wt% (or 0.025 wt%) in the cuvette. The oil drops of (8.5 ± 0.2) l were dispensed via a hook‐shaped dispensing tip into the microgel dispersion within a few seconds (rising drop method). In this study, it used two configurations: rising drop and pendant drop. The rising drop technique (dodecene drop in microgel dispersion) was used instead of the pendant drop (microgel dispersion drop in dodecene) for two reasons: First, the acoustic pressure using a hydrophone could only be done in the aqueous microgel dispersion. Second, eventual depletion effects were avoided due to the large volume of the outer phase. The pendant drop method was used to study the effects of temperature, specially at higher microgel concentrations. The rising drop method was not applicable here because the turbidity resulting from the microgels dispersion going through VPT hinders the optical measurements of interfacial tension. Additionally, the pendant drop method was more resistant to drop rip‐off in long term measurements. Furthermore, the influence of ultrasound on the equilibrium interfacial tension was studied by dispensing drops of microgel dispersion (0.5 wt%, 4.5 ± 0.2 l) via a straight tip in the cuvette filled with 1 ml 1‐dodecene (pendant drop method). Interfacial tension values were obtained every second (or every two seconds for longer measurements) for at least 30 min or until the drop ripped of. The temperature was controlled by placing the cuvette in a temperature cell connected to a thermostat. Inside the cuvette, the temperature was measured with a thermo‐couple with a PT‐100 with an uncertainty of ±0.5 °C. Continuous actuation of the liquid in our experiments does not increase the liquid temperature by more than 2 °C.

## Conflict of Interest

The authors declare no conflict of interest.

## Supporting information

Supporting Information

## Data Availability

The data that support the findings of this study are available from the corresponding author upon reasonable request.
