# Supplementary material for: Ultrasound‐Induced Adsorption of Acousto‐Responsive Microgels at Water–Oil Interface
Source: Adv Sci (Weinh). 2023 Dec 13;11(5):2305395. doi: 10.1002/advs.202305395 (PMC10837341; doi:10.1002/advs.202305395)
Supplement: Supplementary file 1 — Supporting Information [file ADVS-11-2305395-s001.pdf]

ADVANCED SCIENCE
Open Access

Supporting Information

for *Adv. Sci.*, DOI 10.1002/advs.202305395

Ultrasound-Induced Adsorption of Acousto-Responsive Microgels at Water–Oil Interface

Sebastian Stock, Luca Mirau, Matthias Rutsch, Sonja Wismath, Mario Kupnik,
*Regine von Klitzing and Amin Rahimzadeh**

Supporting Information: Ultrasound-induced adsorption of acousto-responsive microgels at oil-water interface

Sebastian Stock,† Luca Mirau,† Matthias Rutsch,‡ Sonja Wismath,‡ Mario Kupnik,‡ Regine von Klitzing,† and Amin Rahimzadeh*,†

†*Soft Matter at Interfaces, Department of Physics, Technische Universität Darmstadt, Hochschulstraße 8, 64289 Darmstadt, Germany*

‡*Measurement and Sensor Technology, Technische Universität Darmstadt, Merckstraße 25, 64283 Darmstadt, Germany*

E-mail: Amin.Rahimzadeh@pkm.tu-darmstadt.de

S1 Verification of MG cleaning

The MGs were cleaned initially by dialisys (10 days, against 50L) and subsequently by centrifugation and redispersion. Figure S1 shows the verification of the cleanliness. What is referred to as cleaning step 0x is corresponding to a MG dispersion that was only cleaned via dialysis.

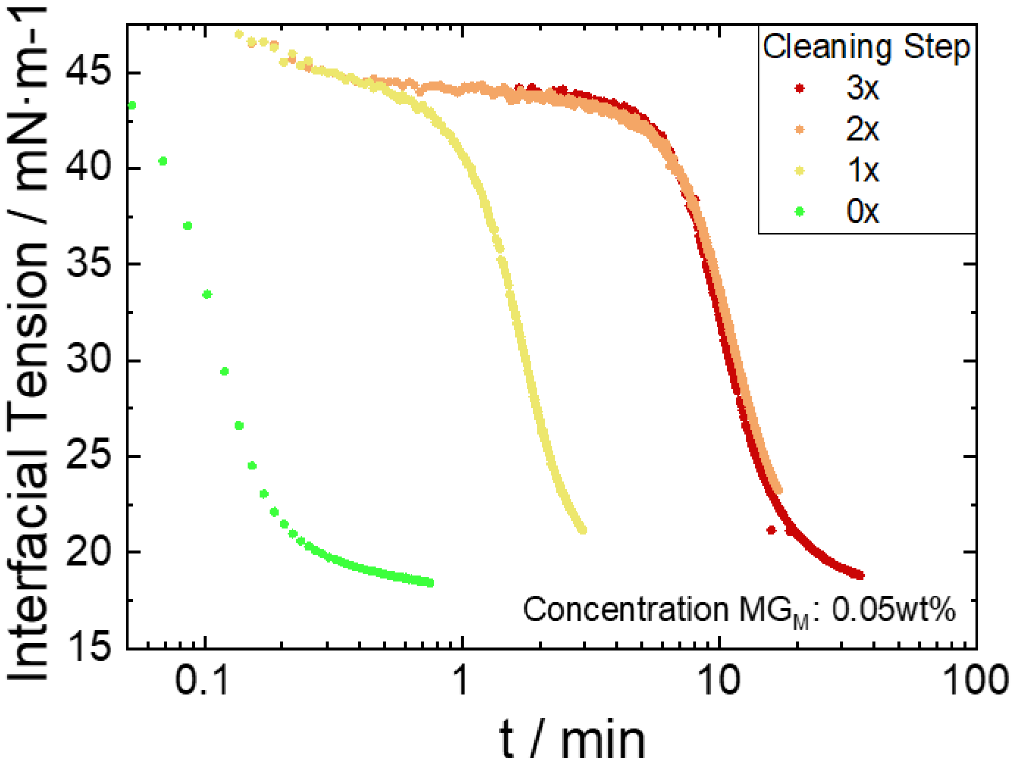

Figure S1: Verification of successful cleaning with centrifugation and subsequent redispersion of MG (5 mol% CL). The green data corresponds to the MG dispersion only cleaned with dialysis. By subsequent cleaning via centrifugation slows the adsorption kinetics. After the impurities are removed, additional cleaning has no effect on the adsorption kinetics (see orange (2x) and red data (3x)).

S2 Setup

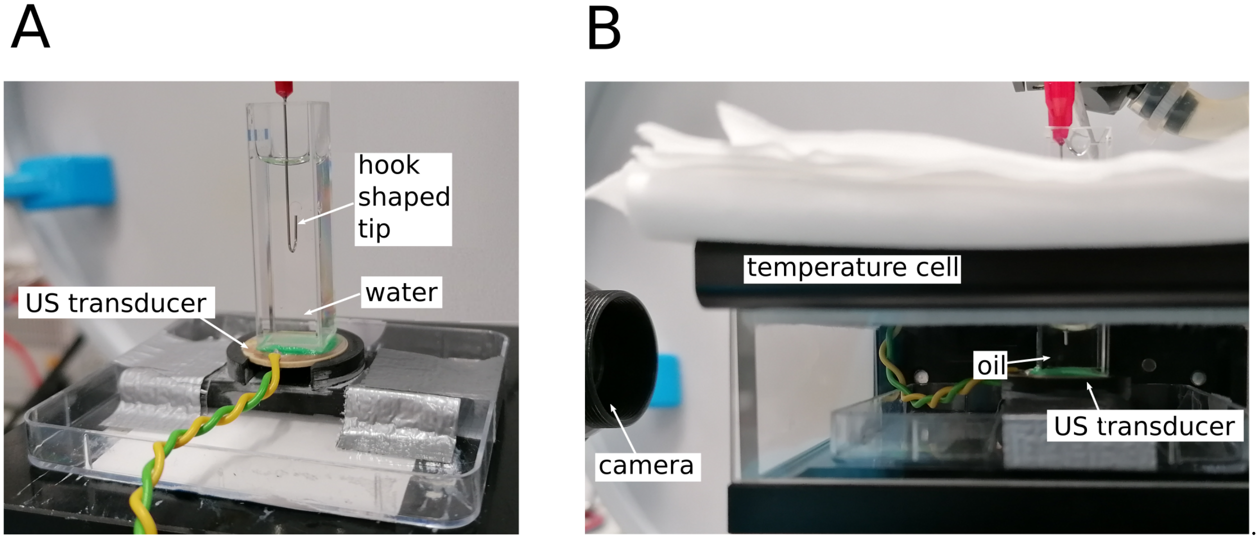

Figure S2: Pictures of the implementation of the US transducer into the drop shape analyzation setup. (A) Measurements at room temperature were performed without a temperature cell. Measurements of the interfacial tension between a oil drop and an aqueous MG dispersion were realized with a hook shaped tip. (B) For measurements at an elevated temperature a temperature cell was used.

S3 Characterization of the MG samples

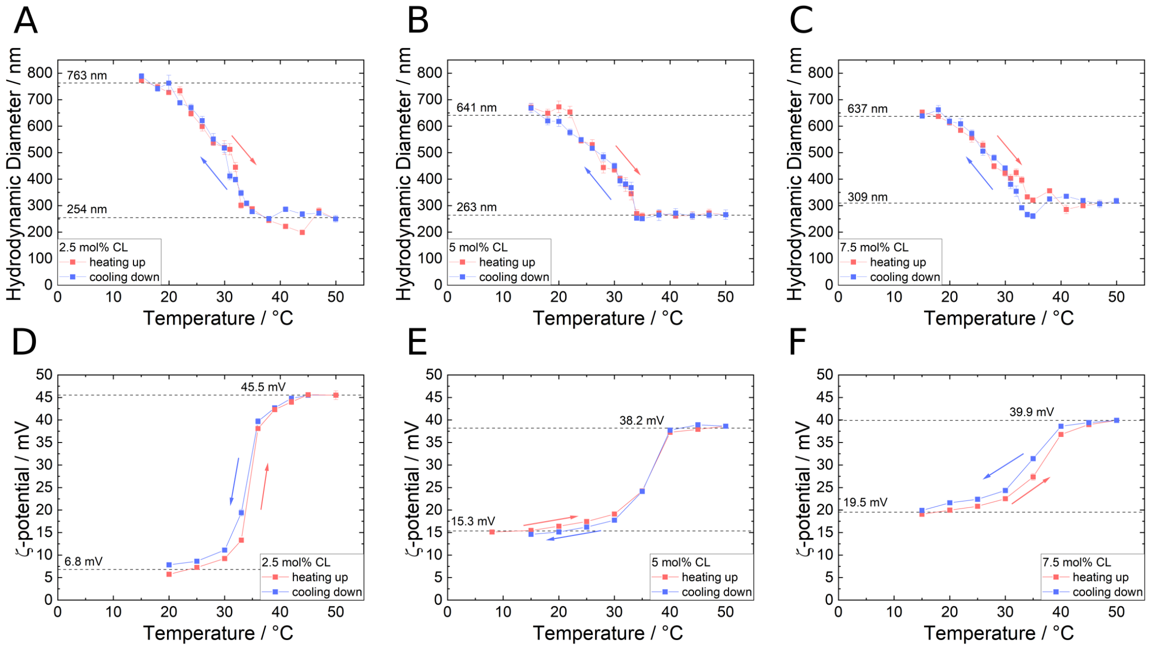

Figure S3: Measured Volume Phase Transition (VPT) behavior of the applied MGs with different cross-linking density. (A-C) Hydrodynamic diameter in dependence of the temperature. All MGs go through a VPT at around 32 °C. (D-F) Corresponding measured ζ-potential. During the VPT the MGs increase their charge. The curves for MGs with 5 mol% CL were reproduced with the permission of the Royal Society of Chemistry.
